# Strategic Acyl Carrier Protein Engineering Enables Functional Type II Polyketide Synthase Reconstitution In Vitro

**DOI:** 10.1021/acschembio.4c00678

**Published:** 2025-01-02

**Authors:** Kevin Li, Yae In Cho, Mai Anh Tran, Christoph Wiedemann, Shuaibing Zhang, Rebecca S. Koweek, Ngọc Khánh Hoàng, Grayson S. Hamrick, Margaret A. Bowen, Bashkim Kokona, Pierre Stallforth, Joris Beld, Ute A. Hellmich, Louise K. Charkoudian

**Affiliations:** † Department of Chemistry, Haverford College, Haverford, Pennsylvania 19041, United States; ‡ Faculty of Chemistry and Earth Sciences, Institute for Organic Chemistry and Macromolecular Chemistry, Friedrich Schiller University Jena, 07743 Jena, Germany; § Department of Paleobiotechnology, Leibniz Institute for Natural Product Research and Infection Biology, Hans Knöll Institute, 07745 Jena, Germany; ∥ Department of Microbiology & Immunology, Center for Advanced Microbial Processing, Institute for Molecular Medicine and Infectious Disease, Drexel University College of Medicine, Philadelphia, Pennsylvania 19104, United States; ⊥ Cluster of Excellence Balance of the Microverse, Friedrich Schiller University Jena, 07743 Jena, Germany; # Center for Biomolecular Magnetic Resonance (BMRZ), Goethe University, 60629 Frankfurt, Germany

## Abstract

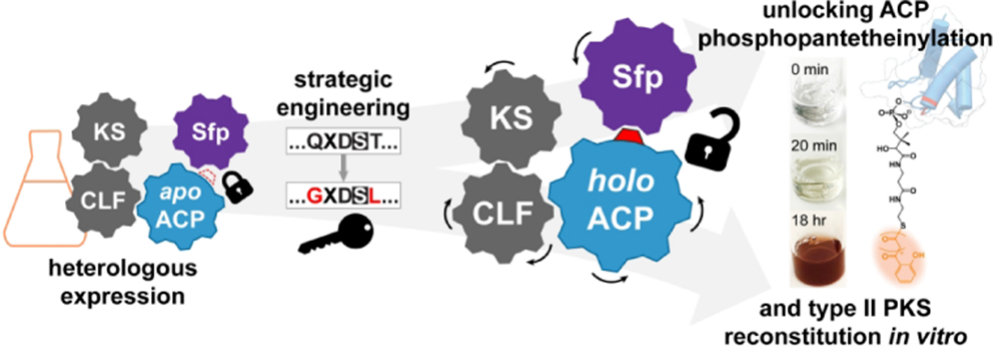

Microbial polyketides represent a structurally diverse class of secondary metabolites with medicinally relevant properties. Aromatic polyketides are produced by type II polyketide synthase (PKS) systems, each minimally composed of a ketosynthase-chain length factor (KS-CLF) and a phosphopantetheinylated acyl carrier protein (*holo*-ACP). Although type II PKSs are found throughout the bacterial kingdom, and despite their importance to strategic bioengineering, type II PKSs have not been well-studied *in vitro*. In cases where the KS-CLF can be accessed via *E. coli* heterologous expression, often the cognate ACPs are not activatable by the broad specificity *Bacillus subtilis* surfactin-producing phosphopantetheinyl transferase (PPTase) Sfp and, conversely, in systems where the ACP can be activated by Sfp, the corresponding KS-CLF is typically not readily obtained. Here, we report the high-yield heterologous expression of both cyanobacterial *Gloeocapsa* sp. PCC 7428 minimal type II PKS (gloPKS) components in *E. coli*, which allowed us to study this minimal type II PKS *in vitro*. Initially, neither the cognate PPTase nor Sfp converted gloACP to its active *holo* state. However, by examining sequence differences between Sfp-compatible and -incompatible ACPs, we identified two conserved residues in gloACP that, when mutated, enabled high-yield phosphopantetheinylation of gloACP by Sfp. Using analogous mutations, other previously Sfp-incompatible type II PKS ACPs from different bacterial phyla were also rendered activatable by Sfp. This demonstrates the generalizability of our approach and breaks down a longstanding barrier to type II PKS studies and the exploration of complex biosynthetic pathways.

## Introduction

The promise of harnessing microbial biosynthetic machineries to gain sustainable access to structurally complex molecules with diverse medicinal properties has inspired decades of research.^1,2^ Polyketides represent a class of natural products with a particularly strong track record for benefiting human health. Landmark studies in the 1990s revealed that polyketide synthases (PKSs) are encoded by biosynthetic gene clusters (BGCs) and act as multifunctional protein assemblies responsible for the microbial biosynthesis of polyketides.^3,4^ In type I PKSs, all protein components required for substrate recognition, activation, transfer and release are organized in a single-chain multidomain protein complex.^5^ In contrast, the components of type II PKSs are expressed separately as monofunctional entities, thereby presenting unique opportunities for strategic engineering.^6^ Aromatic polyketides produced by type II PKSs are important targets for such efforts due to their widespread use as anticancer agents (*e.g.,* doxorubicin) and antibiotics (*e.g.,* tetracycline).^7^

In a minimal aromatic type II PKS, only two essential components are required: a ketosynthase-chain length factor (KS-CLF) and an acyl carrier protein (ACP), which serves to tether the growing polyketide chain and acetate-based building blocks.^7^ This minimal PKS catalyzes the decarboxylative Claisen-like condensations that convert acetate-based building blocks into a nascent β-ketoacyl intermediate.^7^ Once an ACP-bound β-ketoacyl intermediate reaches its programmed length (in part directed by the KS-CLF), it is transformed into the target natural product by a team of accessory enzymes.^7^

The discrete nature of type II PKS domains have made their *in vitro* functional reconstitution the focus of numerous research efforts.^6^ However, attempts to obtain intact KS-CLF enzymes through heterologous expression in fast-growing and easily accessible hosts such as *Escherichia coli* have been challenging, often because of lack of protein production or structural integrity.^8,9^ This has severely limited access to KS-CLFs, making aromatic type II PKS research highly reliant on slow-growing and often difficult to genetically manipulate actinomycete hosts.^10−24^ To address this problem, the focus recently shifted toward KS-CLFs from underexplored phyla inferred to be more closely related to the *E. coli* host than the actinomycete counterparts and thus presumed to be better suited for heterologous expression.^25 −27^ Indeed, numerous cases of the successful heterologous expression of non-actinomycete type II PKS KS-CLFs in *E. coli* have been described,^28−31^ but often without obtaining the cognate ACP in its active “*holo*” form.^28,30,31^ ACP activation occurs post-translationally with a phosphopantetheinyl transferase (PPTase) that covalently attaches a coenzyme A (CoA)-derived 4′-phosphopantetheine (Ppant) arm to a conserved serine residue on the *apo*-ACP.^32^ The Ppant arm of the *holo*-ACP then acts as a molecular tether to shuttle the building blocks and polyketide intermediates through the PKS during biosynthesis. In the laboratory, the native or engineered^33^ PPTase from *Bacillus subtilis* involved in surfactin biosynthesis (Sfp) has typically been used to activate carrier proteins from diverse families including PKSs, fatty acid synthases (FASs) and non-ribosomal peptide synthetases.^32,34^ However, so far there has been limited success using Sfp to activate ACPs in high yields from non-actinomycete type II PKS systems.^6,28,30^

Together, these two issues present a road block for the functional reconstitution of aromatic-synthesizing type II PKSs, i.e., for systems where the ACP can be activated by Sfp, the KS-CLF is typically not readily obtained in high yields and where the KS-CLF can be accessed via *E. coli* heterologous expression, the cognate ACPs could not be activated by Sfp.^28,30^

Here, to overcome these difficulties, we strategically engineered the cyanobacterial *Gloeocapsa* sp. PCC 7428 type II PKS (gloPKS). We were particularly interested in this system because the organism is culturable under laboratory conditions^35^ and thus presents a future opportunity to compare what we learn from *in vitro* studies to the native context. Although high yields for both the gloKS-CLF and the cognate *apo*-gloACP from *E. coli* heterologous expression could be obtained, gloACP could not readily be converted to the active *holo*-form by Sfp, similar to observations by others.^28,30^ By comparing related Sfp-compatible and -incompatible ACP sequences, we identified two residues important for Sfp activation. Mutagenesis of these residues in gloACP did not perturb ACP structure and enabled highly efficient phosphopantetheinylation by Sfp. This minimal mutagenesis strategy was conferrable to other Sfp-incompatible ACPs from other bacterial phyla, thus demonstrating the generalizability of the approach. In the case of the gloPKS, the *holo*-ACP could be loaded with a salicyl-priming unit and malonyl substrates through enzyme-facilitated reactions. Together with the heterologously expressed and purified gloKS-CLF, this allowed us to study the minimal type II PKS and generate a range of aromatic products *in vitro*. This study thus represents an important step toward the use of type II PKS for future bioengineering approaches.

## Results and Discussion

### *Gloeocapsa* sp. PCC 7428 Minimal Type II PKS Components Can Be Heterologously Expressed in *E. coli* with High Yields

Previous bioinformatic and phylogenetic analyses suggested that *Gloeocapsa* sp. PCC 7428 harbors a type II PKS BGC (Figure S1) containing a *ks-clf* gene that is inferred to be more closely related to the *E. coli* FAS KS-encoding gene *fabF* than other actinobacterial *ks-clf*s.^25,26^ This indicated that gloKS-CLF might be a promising candidate for heterologous expression in *E. coli*.^28,30^ Moreover, the glo *ks* and *clf* genes are transcriptionally coupled in the native host, a feature that could be important for the successful *E. coli* expression of the KS-CLF heterodimer.^8^ Indeed, we found that compared to the relatively slow-growing actinobacterial host, gloKS-CLF could be obtained in roughly 25-fold greater yields, i.e., with >20 mg/L purified protein from *E. coli* BL21(DE3) cells when expressed under a single promoter (Figures S2 and S3). Likewise, the cognate gloACP could be obtained in high amounts (∼17 mg/L) and was present exclusively in its *apo*-form upon purification (Figures S2 and S4).

### *Apo*-GloACP is Not Readily Converted to Its Active *Holo*-Form

Typically, ACPs are obtained in their *holo*-form through either the heterologous expression in *E. coli* BAP1 cells, a genetically engineered derivative of the BL21 (DE3) strain carrying background expression of Sfp,^36^ or the *in vitro* reaction of purified ACP with recombinant *B. subtilis* Sfp.^33,37,38^ An Sfp with increased catalytic efficiency and substrate scope, termed Sfp R4–4, is typically used for ACP activation.^33^ We also made use of this variant and refer to it as Sfp throughout the manuscript. Neither expression in *E. coli* BAP1^36^ nor treatment of purified *apo*-ACP with Sfp yielded the phosphopantetheinylated gloACP, as shown by mass spectrometry (Figures S4 and S5). Coexpression of gloACP with its putatively cognate PPTase in *E. coli* also did not lead to the desired production of *holo*-ACP (Figures S6 and S7). Finally, using an *in vitro* approach, we coincubated *apo*-gloACP with purified gloPPTase, produced separately in *E. coli*, where it can be expressed at low levels (Figure S8). Again, this did not yield *holo*-ACP (Figure S9). While it remains unclear whether the heterologously expressed gloPPTase was properly folded, it is notable that even in the presence of the putative CoA:ligase domain from *Gloeocapsa* (salicylate CoA:ligase; gloSCL; Figures S1 and S2) and its cosubstrates (adenosine triphosphate (ATP) and salicylic acid), a strategy that resulted in ACP activation in other systems,^28,31^ the gloACP evaded activation (Figure S10). These data highlight that gloACP activation is not straightforward and represents a barrier to the study of the minimal type II gloPKS.

### Sfp Interacts with GloACP, But Does Not Convert it to Its *Holo-*Form

**Figure 1:**
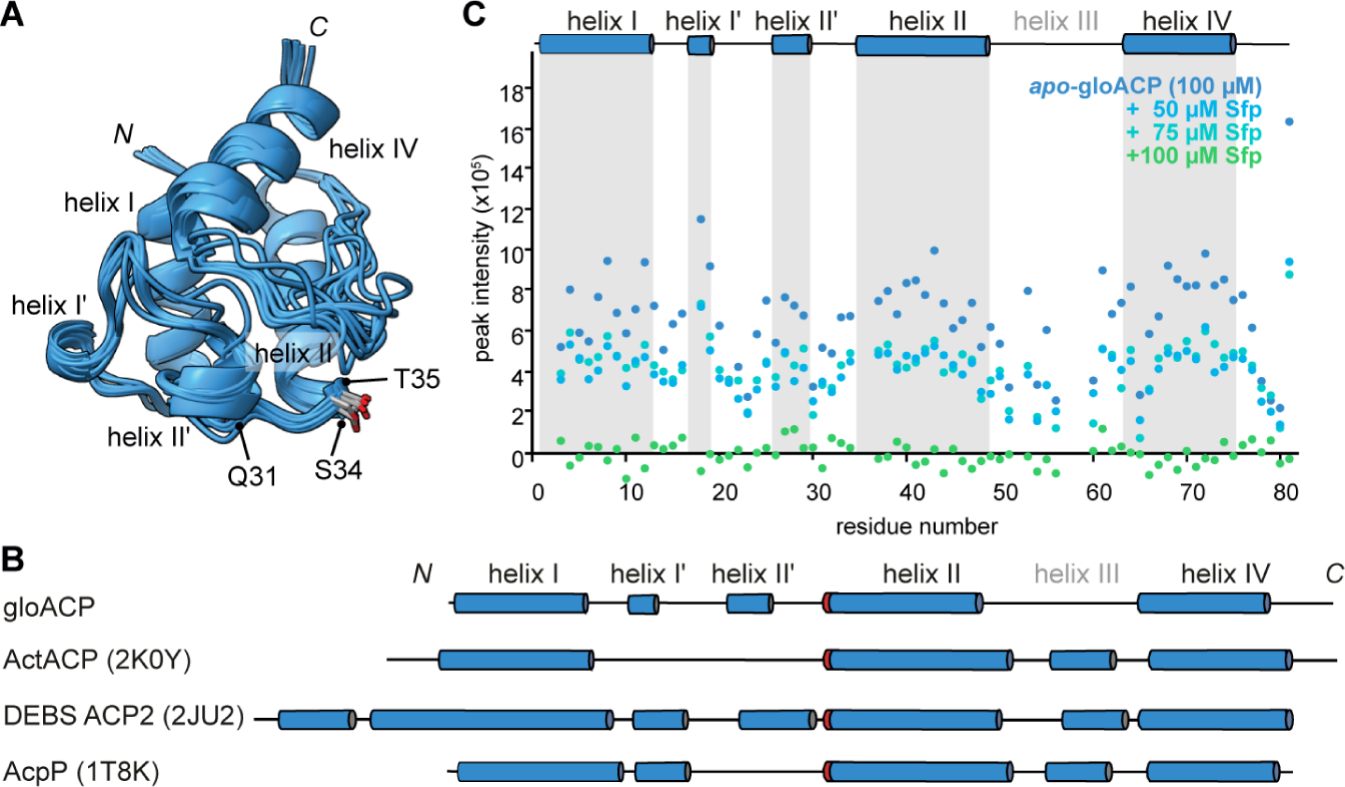
*Gloeocapsa* ACP has a canonical fold and can interact with the *B. subtilis* PPTase Sfp. (A) The ten lowest energy structural models of WT gloACP obtained by CS-Rosetta. Residues Q31 and T35 important for Sfp-based ACP activation, as well as S34, the attachment point of the phosphopantetheine arm are marked. (B) Comparison of topology models for gloACP (based on AlphaFold) and available structures of Sfp-activatable, prototypical ACP domains: a type II PKS ACP (ActACP, PDB: 2K0Y) from *Streptomyces coelicolor*, a type I PKS ACP (DEBS ACP2, PDB: 2JU2) from *Saccharopolyspora erythraea*, and a type II FAS ACP (AcpP, PDB: 1T8K) from *Escherichia coli* (cylinders represent alpha-helices with red marking indicating the serine point of attachment of the Ppant arm. (C) NMR peak intensities of ^15^N-labeled gloACP upon Sfp titration. Severe line broadening at a 1:1 molar ratio (both proteins at 100 μM) indicates tight complex formation (see also Figures S11–S13). Note that helix III found in most carrier proteins is absent in gloACP.

Initially, we wondered whether the inability of Sfp to activate gloACP might be due to structural differences between gloACP and ACPs known to be Sfp-compatible.^32,34^ For a structural characterization of gloACP, we determined the backbone and side chain NMR assignments of the ^13^C, ^15^N-labeled protein (Figure S11) and determined a chemical shift-derived structural model using CS-Rosetta^39^ (Figures 1A and S12). Comparison with available X-ray and NMR structures of Sfp-compatible ACPs from canonical type II FAS (*E. coli* AcpP, PDB: 1T8K), actinobacterial type II PKS (*Streptomyces coelicolor* ActACP, PDB: 2K0Y), and type I PKS (*Saccharopolyspora erythraea*, DEBS ACP2, PDB: 2JU2) systems revealed small structural differences but an overall canonical ACP fold for the *Gloeocapsa* protein (Figure 1B). GloACP differs from AcpP and ActACP by two short helical insertions between helix I and helix II (which we accordingly refer to as helix I′and helix II′), and lacks helix III which is common in other ACPs.^32^ Nonetheless, the overall secondary and tertiary structure arrangements of gloACP and importantly, the position of the serine residue at the *N*-terminus of helix II required for Ppant arm attachment are conserved compared to known Sfp substrates.

Previously, we and others have successfully used NMR chemical shift perturbations to assess transient ACP-protein interactions.^40−42^ Here, we titrated the ^15^N-labeled 11 kDa gloACP with unlabeled 27 kDa Sfp and observed line broadening at substoichiometric ratios, suggesting the formation of a larger molecular weight complex (Figures 1C and S13). At an equimolar ratio, all gloACP signals disappeared, consistent with tight complex formation between gloACP and Sfp. While line broadening thus precludes a residue-specific analysis, this indicates that neither structural differences nor lack of protein interactions are likely to be the decisive cause for the inability of Sfp to activate gloACP.

### Minimal GloACP Sequence Modifications Enable Sfp-Mediated Transformation from *Apo* to *Holo-*Form

**Figure 2:**
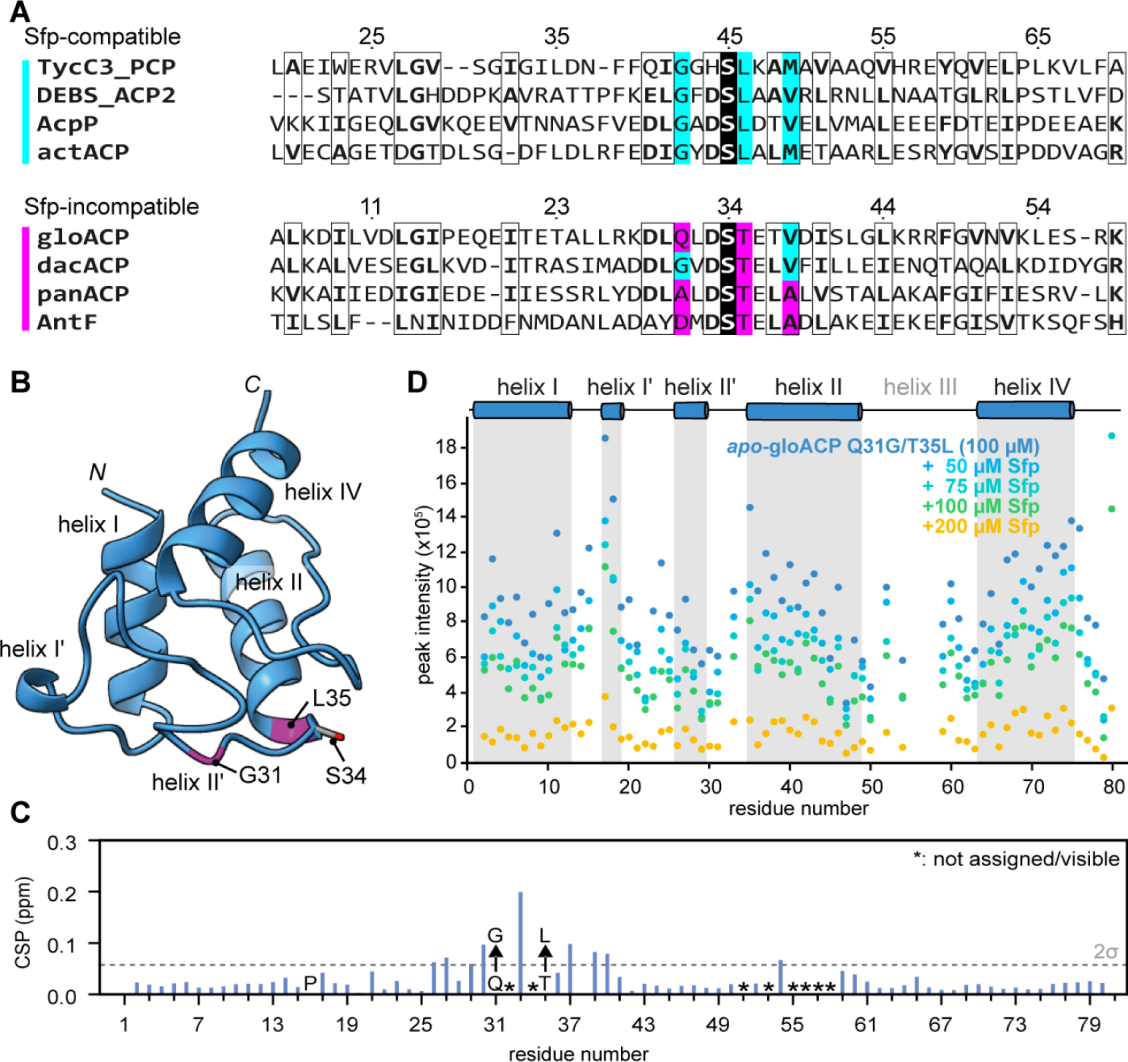
Sfp compatibility can be inferred from ACP sequence, enabling targeted modifications to render ACPs activatable through loosened Sfp interactions. (A) Multiple sequence alignment of carrier proteins known to be modified by Sfp (top four with cyan bar) and non-actinobacterial Sfp-incompatible ACPs (bottom four with magenta bar). Numbering is based on the TycC3_PCP^43^ and gloACP sequences, respectively. GloACP, dacACP, and panACP were used in this study (see main text for details), while AntF is a previously characterized non-actinobacterial ACP from *Photorhabdus luminescens*.^44^ The serine carrying the phosphopantetheine arm is highlighted in black in each sequence. Black boxes represent residues with greater than 70% physicochemical similarity. Based on our and previously published functional data (see main text for details) as well as the sequence alignments, we hypothesized that the highlighted residues (cyan and magenta, respectively) direct Sfp-(in)compatibility. (B) CS-Rosetta model (see Figure 1A) of gloACP^Q31G/T35L^ indicating the position of the two mutations Q31G and T35L. (C) Chemical shift perturbations comparing gloACP WT and the Q31G/T35L double mutant. The proline residue is marked with P and unassigned residues are marked with an asterisk. (D) Relatively higher peak intensity of ^15^N-labeled gloACP^Q31G/T35L^ (100 μM) upon titration with unlabeled Sfp indicates looser interaction compared to WT gloACP (see Figures 1C and S13). Note that the ACP concentration was kept constant and accordingly, increases in linewidth stem from Sfp binding, not sample dilution.

To overcome the inability of Sfp to activate the carrier protein, we looked at the tyrocidine A synthetase peptidyl carrier protein (TycC3_PCP) from *Bacillus brevis*, which has been studied in complex with Sfp.^43^ TycC3_PCP residues G42, L46, and M49 were found to be crucial for phosphopantetheine transfer by Sfp. A multiple sequence alignment of Sfp-compatible and incompatible carrier proteins suggests that two out of the three positions identified in TycC3_PCP may also affect Sfp (in)compatibility with gloACP (Figures 2A and S14). In Sfp-compatible ACP sequences, the position of the glycine and a hydrophobic residue analogous to TycC3_PCP L46 are generally preserved (marked in cyan in Figure 2A). In contrast, these residues are more varied in Sfp incompatible ACPs with *e.g.,* gloACP Q31 and T35 (marked in magenta in Figure 2A). These residues flank the serine important for phosphopantetheinylation at the *N*-terminus of helix II (Figure 1A). To evaluate whether these residues play a role in Sfp-based activation, we generated single and double gloACP mutants emulating the TycC3_PCP sequence, i.e., gloACP^Q31G^, gloACP^T35L^ and gloACP^Q31G/T35L^. All gloACP mutants could be heterologously expressed in *E. coli* and purified in their *apo*-form (Figures S15 and S17). As described above, WT gloACP was not phosphopantetheinylated by Sfp *in vitro* (Figure S5). Likewise, no phosphopantetheinylation for gloACP^Q31G^ and minimal conversion of gloACP^T35L^ (∼5% conversion after 16h) by Sfp were observed by mass spectrometry (Figure S18). In contrast, gloACP^Q31G/T35L^ was successfully converted to its *holo*-form, yielding ∼95% conversion (Figure S16). Interestingly, this gain in Sfp-compatibility for gloACP^Q31G/T35L^ was not due to structural changes compared to the WT ACP, as indicated by the minimal chemical shift differences of the two proteins, but rather due to an apparent loosening of the interaction with Sfp as indicated by the lower degree of line broadening (Figures 1C and 2D).

To reflect the presence of other hydrophobic residues in some of the available ACP sequences at the position homologous to gloACP T35, we also generated the Q31G/T35I double mutant. Sfp successfully phosphopantetheinylated this variant, albeit with lower efficiency than the Q31G/T35L double mutant (∼30% conversion, Figure S19). Importantly, this shows that minimal sequence modifications are sufficient to convert an Sfp-incompatible into a compatible ACP.

### Minimal Sequence Adaptations Present a General Route to Render Previously Inaccessible ACPs Sfp-Compatible

Having identified the two relevant residues for the cyanobacterial gloACP activation by Sfp, we next evaluated the generalizability of this approach for other ACPs. To this end, we selected type II PKS ACPs from *Delftia acidovorans* (dacACP) and *Paenibacillus* sp. FSL R7–277 (panACP) (Figure 2A), representing the proteobacteria and firmicutes phyla.

Both dacACP and panACP contain a threonine residue at positions analogous to gloACP T35 and TycC3_PCP L46 (Figure 2A). According to our hypothesis, these residues should interfere with Sfp-compatibility. In addition, panACP contains two alanine residues (A31, A38) at positions analogous to TycC3_PCP G42 and M49, that we would thus deem to hamper Sfp-compatibility. In contrast, we would predict the respective residues in dacACP, i.e., G31, V38, to enhance Sfp-compatibility, suggesting that Sfp may retain some ability to modify dacACP.^43^ Both WT dacACP and panACP were purified from *E. coli* in their *apo*-form (Figures S20 and S22). In agreement with the expectation for a tapered ability of either ACP to become activated by Sfp, we saw that WT panACP was not phosphopantetheinylated, while WT dacACP was phosphopantetheinylated to a very low degree (<5%, Figures S20–23). Next, we aimed to transform panACP and dacACP into fully Sfp-compatible proteins. Analogous to the gloACP^Q31G/T35L^ variant, we generated two constructs emulating the TycC3_PCP sequence expected to enable phosphopantetheinylation, i.e., dacACP^T43L^ and panACP^A30G/T34L/A37V^. Both variants were successfully purified from *E. coli* in their *apo*-form (Figures S24 and S26) and found to be fully phosphopantetheinylated by Sfp (Figures S25 and S27). This underscores the importance of a nonpolar residue in a position analogous to TycC3_PCP L46 for efficient phosphopantetheinylation by Sfp. Importantly, these findings also suggest that Sfp (in)compatibility can be directly predicted from the ACP sequence and minimal protein engineering can endow type II PKS ACPs with the ability to be modified by Sfp. Together with the increased access to KS-CLFs via *E. coli* heterologous expression, this opens the door for the functional reconstitution of hitherto inaccessible type II PKSs *in vitro*.

### The Reconstituted Minimal GloPKS Produces Polyketides In Vitro

**Figure 3:**
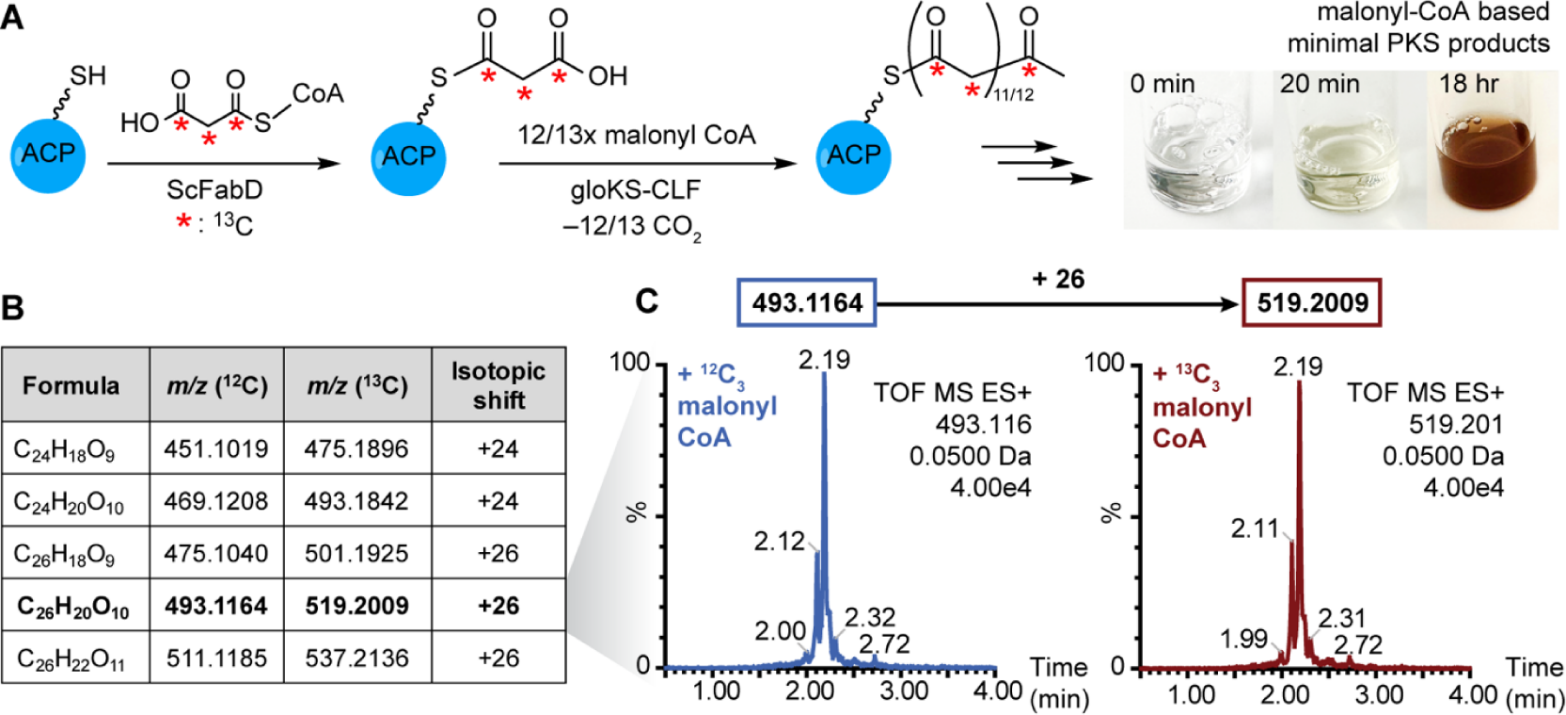
Reconstitution of *holo*-gloACP^Q31G/T35L^and gloKS-CLF yields a functional minimal type II PKS *in vitro*. (A) Proposed *in vitro* reaction scheme of gloPKS polyketide production by the minimal gloPKS components and color switch of reaction mixture after addition of ScFabD and malonyl-CoA to *holo*-gloACP and gloKS-CLF. (B) Chemical formulas and experimental *m*/*z* values (^12^C and ^13^C) of putative precursor polyketide metabolites produced by the minimal gloPKS with malonyl-CoA. (C) Extracted ion chromatograms of the putative metabolite C_26_H_20_O_10_. ^13^C_3_-malonyl-CoA supplementation leads to a mass shift of +26 amu in both cases (for additional extracted ion chromatograms of putative precursor polyketides see Figure S31).

After gaining access to the quantitative modification of gloACP by Sfp, we aimed to functionally reconstitute the minimal type II gloPKS composed of ACP and KS-CLF *in vitro* (Figure 3). We first assessed whether the Ppant arm of *holo*-gloACP^Q31G/T35L^ can be acylated with a malonyl group, a critical step in the formation of a growing polyketide chain. The majority of ACPs studied to date require malonyl-CoA:acyl carrier protein transacylases (MCATs) to catalyze this process.^45,46^ However, in select cases, MCATs are not required and ACPs can “self-malonylate”.^13,47^ Upon incubation with malonyl CoA, a low degree of self-malonylating activity was observed for *holo*-gloACP^Q31G/T35L^ (Figure S28). However, for full conversion from *holo-* to malonyl-gloACP^Q31G/T35L^, coincubation with MCATs from either *Streptomyces coelicolor* (ScFabD) or *E. coli* (EcFabD) was required (Figures S29 and S30).

Next, we explored whether the minimal gloPKS can produce polyketides *in vitro*. This requires the productive interaction of both heterologously produced protein units to mediate the two central polyketide biosynthesis reactions: chain transfer and elongation. Consistent with the formation of polyaromatic products, mixing gloKS-CLF, *holo*-gloACP^Q31G/T35L^, ScFabD and malonyl CoA resulted in in the generation of compounds and a concomitant color change from clear to orange/brown (Figure 3A, right). Isolation of the colored molecules and analysis by high-resolution mass spectrometry showed the presence of compounds with molecular formulas of C_24_H_18_O_9_ (*m*/*z* = 451), C_24_H_20_O_10_ (*m*/*z* = 469), C_26_H_18_O_9_ (*m*/*z* = 475), C_26_H_20_O_10_ (*m*/*z* = 493), and C_26_H_22_O_11_ (*m*/*z* = 511) (Figures 3B and S31). Feeding experiments with ^13^C_3_-malonyl-CoA resulted in isotopic shifts of 24-amu (451, 469) and 26-amu (475, 493, 511), suggesting the production of dodecaketides and tridecaketides via condensation of 12 and 13 molecules of malonyl-CoA, respectively, by the minimal gloPKS *in vitro* (Figure 3C). The successful activation of the PKS biosynthetic machinery led to the production of a highly complex product mixture, precluding the purification of individual compounds in sufficient quantities for additional structural characterization.

### Additional Enzymes and Substrates Expand the Scope of the Minimal GloPKS

**Figure 4:**
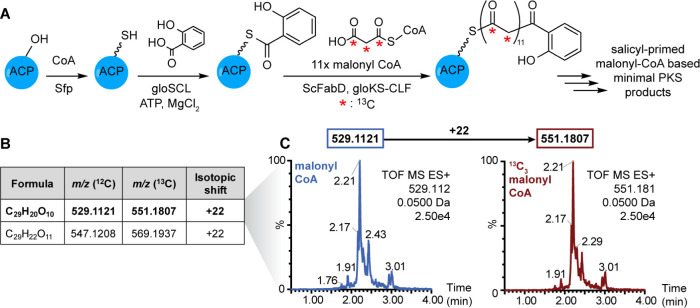
Salicyl priming expands the product profile of the reconstituted minimal gloPKS. (A) Proposed *in vitro* gloPKS polyketide production scheme. Red stars represent carbon atoms derived from malonyl-CoA and anticipated to be labeled during ^13^C_3_-malonyl-CoA isotope experiments. (B) Chemical formulas and experimental *m*/*z* values (^12^C and ^13^C) of putative precursor polyketide metabolites obtained from LC-MS experiments. (C) Extracted ion chromatograms of the putative metabolite C_29_H_20_O_10_. Upon ^13^C_3_-malonyl-CoA supplementation, a mass shift of +22 amu is observed (for additional extracted ion chromatograms of putative precursor polyketide products see Figure S33).

After obtaining a functional minimal gloPKS *in vitro*, we wondered whether its product scope can be expanded through addition of alternative substrates. The gloPKS BGC shows similarities to the thermorubin-producing PKS from *Laceyella sacchari*, suggesting that the gloPKS can be primed by salicylic acid (Figure S1 and Table S1).^48^ Indeed, we observed that the addition of ATP and the heterologously expressed cognate salicylate:CoA ligase (gloSCL) allowed priming of the *holo*-gloACP^Q31G/T35L^ with salicylic acid (Figure S32). In the presence of the gloKS-CLF, salicyl-gloACP^Q31G/T35L^ underwent multiple rounds of chain extension with the malonyl-CoA building block, resulting in C29 salicyl-primed products (Figure 4A,B). High-resolution mass spectrometry revealed the formation of products consistent with the formulas C_29_H_20_O_10_ and C_29_H_22_O_11_, i.e., *m*/*z* 529 and 547 [M + H]^+^. Feeding of ^13^C_3_-labeled malonyl-CoA led to a mass shift of 22 amu for both products (Figures 4B,C, and S33), indicating the incorporation of 11 malonyl-CoA units. This is consistent with the thermorubin biosynthetic pathway, which affords a 29-carbon polyketide backbone from seven carbons derived from salicylate and 22 carbons derived from malonyl-CoA.^48,49^

Importantly, these results show that the engineered *holo*-gloACP^Q31G/T35L^ variant remains a functional unit within an expanded PKS roster that includes pathway priming components. Moreover, the distinct products manufactured in the presence versus absence of salicylic acid priming supports the concept that core type II PKS biosynthetic components can be shuffled to direct the chain lengths of polyketides produced.

## Conclusions

Historially, the inability to obtain minimal type II PKS components for *in vitro* sudies has limited the bioengineering of these systems. Access to KS-CLFs and *holo-*ACPs opens the door for investigations focused on substrate specificity and screening, kinetics, mechanisms, combinatorial biosynthesis, structural analyses and more. Here, we have shown that ACP compatibility with the PPTase Sfp, and by extension ACP activation for the functional reconstitution of type II PKSs, can be directly inferred from the ACP sequence. Minimal protein engineering of the ACP enables the efficient transformation of heterologously produced non-actinomycete *apo*-ACPs into their active *holo*-form by Sfp. This, together with improved access to KS-CLFs via the *E. coli* heterologous expression of non-actinomycete target genes as outlined in the recent literature^28−31^ and highlighted in the current work, allowed to reconstitute a functional minimal type II PKS *in vitro*. Using the minimal gloPKS, we saw that this system can make use of diverse priming molecules to create a large set of aromatic products. However, it remains unclear whether the product diversity observed *in vitro* aligns with the *in vivo* output profile. It is tempting to conclude that unknown crowding factors or accessory proteins regulate the product synthesis specificity in native systems. However, we also speculate that there could be exceptions to the “one cluster one product” paradigm in which the ability to incorporate varying priming molecules could be a feature of synthases in bacteria, as these organisms are exposed to fast changing environmental demands (*e.g.,* nutrient fluctuations or encounters with competition). The *Gloeocapsa* type II PKS is particularly well-suited to follow up on important questions that require bridging *in vitro* and *in vivo* aspects. The organism is culturable under laboratory conditions^35^ and, as we have shown in the present work, the gloPKS components can be heterologously produced for *in vitro* investigations.

The workflow presented here provides a general and easy route to *holo*-ACP from biosynthetic machineries in diverse phyla that were hitherto inaccessible. This will enable future exploits into combinatorial biosynthesis schemes with reconstituted type II PKS domains. It is intriguing to point out that an apparent loosening of protein binding enabled efficient phosphopantetheinylation of the ACP by Sfp. This suggests that the lack of activation of the WT gloACP to *holo-*form is due to a lack of catalysis rather than lack of binding. These findings showcase another example for the importance of transient, short-lived interactions in functional PKSs.^42,50^ The interplay between transient domain interactions and biosynthetic steps, as well as how synthase components tune the binding affinities necessary to maintain synthase activity and fidelity, represent important considerations in future type II PKS reconstitution work and will be the subject of future work. Enhancing intramolecular contacts that, as we have seen, can be detrimental to function, could thus guide the development of inhibitors targeting bacterial PPTases implicated in the virulence factors of common pathogens, further expanding the impact of such type II PKS research.^34,51^

## Methods

### Native Organism Culturing and DNA Isolation

*Gloeocapsa* sp. PCC 7428 (ATCC 29159) was purchased as a liquid culture from the American Type Culture Collection (ATCC) and inoculated in 30 mL of BG-11 liquid medium (ATCC medium 616).^35^ Cultures were grown at RT with indirect light exposure for 6–7 weeks with aeration and no shaking. Genomic DNA was isolated following the protocol described in Saha *et al*.^52^

### Molecular Cloning

*E. coli* DH5α competent cells were used for all cloning experiments. AntiSMASH 5.0 was used to identify genes corresponding to *Gloeocapsa* sp. PCC 7428 (ID: CP003646) ACP, KS-CLF, PPTase, and SCL genes.^53^ Each gene was amplified from purified genomic DNA using primers listed in Table S2 and standard Q5 High-fidelity DNA polymerase. For genes corresponding to ACPs from *Paenibacillus* sp. FSL R7–277 and *Delftia acidovorans*, codon optimized DNA was purchased from Twist Bioscience and amplified (see Table S2 for DNA sequences). Gel-purified amplimers were inserted into linearized pET28a vector backbone between NdeI and EcoRI cleavage sites (except for the malonyl-CoA synthetase, MatB, which was inserted between NdeI and XhoI cleavage sites) via Gibson assembly^54^ using an NEB Q5 high-fidelity 2X master mix kit (for plasmids, see Table S2). The expression plasmid for EcFabD was provided as a gift from the Campopiano Research group at University of Edinburgh, the plasmid for ScFabD was purchased from Gene Universal. Plasmid sequences were confirmed by whole plasmid sequencing (Plasmidsaurus).

### Multiple Sequence Alignment (MSA)

Amino acid sequences of the carrier proteins of interest were aligned using Clustal Omega and ESPript 3.0.^55,56^

### Site Directed Mutagenesis

Mutant plasmids of the ACPs were obtained using the NEB Q5 site directed mutagenesis kit following the manufacturer’s protocol. In brief, amplification was performed on the mixture of Q5 Hot Start High-Fidelity 2X Master Mix, 10 μM forward primer, 10 μM reverse primer, template DNA (1–25 ng of the WT ACP plasmid), and nuclease-free water. Kinase, ligase and DpnI (KLD) treatment was conducted by mixing the PCR product, 2X KLD reaction buffer, 10X KLD enzyme mix, and nuclease-free water, followed by incubation at RT for 5 min. The KLD mix was transformed into *E. coli* DH5α competent cells and plated onto LB agar supplemented with 50 μg/mL kanamycin (Kan).

pGloACP was amplified with four distinct primer sets to create the gloACP mutants gloACP^Q31G/T35L^, gloACP^Q31G/T35I^, gloACP^Q31G^, and gloACP^T35L^. Vectors pPanACP and pDacACP encoding WT panACP and dacACP, respectively, were used to create mutants panACP^A30G/T34L/A37V^ and dacACP^T43L^. All primers and insert sequences are provided in Table S2.

### Protein Expression and Purification

All proteins were purified according to previously reported protocols.^57^ In brief, plasmids were transformed into competent *E. coli* BL21(DE3) cells or, to aim to express *holo*-gloACP, into BAP1 cells^36^ as specified in the main text. Seed cultures were prepared by inoculating a single bacterial colony in 10 mL of LB supplemented with 50 μg/mL Kan and incubating overnight at 37 °C with shaking for 18 h. Production cultures (1 L LB supplemented with 50 μg/mL Kan) were inoculated with the 10 mL overnight seed culture, incubated at 37 °C and induced with isopropyl β-D-1-thiogalactopyranoside (IPTG, final concentration of 250 μM) once cultures reached an OD_600_ of 0.4–0.6. After overnight incubation (18 °C, 200 rpm), cells were harvested by centrifugation (4 °C, 4424 × *g*, 20 min) and stored at −80 °C until purification. For purification, cell pellets were resuspended on ice using lysis buffer (50 mM sodium phosphate buffer, 10 mM imidazole, 300 mM NaCl, 10% (v/v) glycerol, pH 7.6) and lysed (Microson XL-200 Ultrasonic Processor: 10 × 30 s pulses with 30 s rest in between at 40% amplitude). Cellular debris was removed via centrifugation (4 °C, 17,000 × *g*, 1 h), and the resulting supernatant was transferred to nickel-NTA agarose equilibrated with lysis buffer and mixed for 1–2 h at 4 °C with gentle rocking. The protein-bound resin suspension was transferred into a gravity flow column (Bio-Rad Econo-Pac-chromatography column) equipped with a 30 μM polyethylene bed and allowed to settle before collecting the flow through and washing twice using wash buffer (2 × 100 mL of 50 mM sodium phosphate buffer, 30 mM imidazole, 300 mM NaCl, 10% (v/v) glycerol, pH 7.6). Finally, the His_6_-tagged proteins were eluted with 10 mL of elution buffer (50 mM sodium phosphate buffer, 250 mM imidazole, 100 mM NaCl, 10% (v/v) glycerol pH 7.6) and quantified using UV/vis or bicinchoninic acid (BCA) assays for proteins lacking amino acids with aromatic side chains. All proteins were aliquoted, flash-frozen, and stored at −80 °C. Sodium dodecyl sulfate polyacrylamide gel electrophoresis (SDS-PAGE) and Western Blot were used to confirm protein purity (detailed in Supporting Information Methods, Figures S2 and S8).

### NMR Spectroscopy

Uniformly ^15^N-labeled and ^13^C/^15^N labeled *N*-terminal His_6_-tagged gloACP was expressed in *E. coli* BL21(DE3) gold cells grown in M9 minimal medium^58^ supplemented with 1 g/L ^15^NH_4_Cl and 2 g/L U–^13^C-glucose as the sole nitrogen and carbon sources, respectively, following a spin-down protocol. For ^15^N labeling, 1 g/L ^15^NH_4_Cl was used as the sole nitrogen source and the M9 minimal medium was supplemented with unlabeled glucose (2 g/L) as carbon source. Transformed *E. coli* cells were first grown in 1 L LB medium at 37 °C with shaking at 180 rpm to an OD_600_ = 0.4–0.6 and then centrifuged (25 °C, 5000 × *g*, 10 min). The cell pellets were resuspended in 1 L M9 minimal medium supplemented with 50 μg/mL Kanamycin and incubated for 30 min at 37 °C with shaking at 180 rpm. Protein expression was induced with 312 μM IPTG and cells were grown for 16 h at 18 °C with shaking at 180 rpm. Cells were then harvested via centrifugation (4 °C, 5000 × *g*, 20 min).

Cell pellets were resuspended in 100 mL of 50 mM sodium phosphate buffer (pH 7.6) containing 300 mM NaCl, 10 mM imidazole, 10% (v/v) glycerol, a protease inhibitor mix (1:1000 dilution), and a spatula tip of lysozyme, DNase, and RNase. Cells were lysed by sonication, and the lysate was clarified by centrifugation (4 °C, 10,000 × *g*, 30 min). The supernatant was incubated with pre-equilibrated nickel-NTA beads (2 mL/L of culture) for 1 h on an end-over-end rotator. The mixture was transferred to a gravity column, washed sequentially with 10 column volumes (CV) of 50 mM sodium phosphate buffer (pH 7.6) containing 100 mM NaCl, 10 mM imidazole, and 10% (v/v) glycerol, followed by 10 CV of the same buffer containing 30 mM imidazole. The protein was eluted in five 1 CV steps with 50 mM sodium phosphate buffer (pH 7.6) containing 100 mM NaCl, 250 mM imidazole, and 10% (v/v) glycerol. Protein-containing fractions were identified using a BCA assay, pooled, and concentrated using Sartorius Vivaspin 3000 MWCO filters. Size exclusion chromatography was performed using a HiLoad prep grade 16/60 Superdex 75 column equilibrated with 50 mM sodium phosphate buffer (pH 7.6) containing 100 mM NaCl.

All NMR experiments were conducted at 298 K on Bruker AVANCE III HD 600 MHz spectrometers equipped with cryogenic triple resonance probes (Bruker BioSpin GmbH, Rheinstetten). Triple resonance assignment experiments (HNCO, HN(CA)CO, HNCA, and HNCACB) were recorded using standard Bruker TopSpin pulse sequences for resonance chemical shift assignments. Both wild-type gloACP and gloACP^Q31G/T35L^ were measured at concentrations of 300–410 μM in 50 mM sodium phosphate buffer (pH 7.5) containing 100 mM NaCl, 5% (v/v) *d*_6_-glycerol. Samples were supplemented with 10% D_2_O for field-frequency locking and 0.1 mM 2,2-dimethyl-2-silapentane-5-sulfonic acid (DSS) for chemical shift referencing. The ^1^H signal of DSS was set to 0 ppm for direct referencing and the ^13^C and ^15^N chemical shifts were indirectly referenced according to their magnetogyric ratios.^59^ Data processing was performed using Bruker TopSpin 3.2 or 4.1 software, and analysis of wild-type gloACP, gloACP^Q31G/T35L^, and *holo*-gloACP^Q31G/T35L^ was carried out using CCPN V3.1.^60^ For titration experiments with Sfp R4–4, ^15^N-labeled WT gloACP and ^15^N-labeled gloACP^Q31G/T35L^ were used. NMR samples were prepared by diluting the stock solutions of ^15^N wild-type gloACP or ^15^N gloACP^Q31G/T35L^ (both 270 μM) in the appropriate buffer to achieve a final concentration of 100 μM gloACP or gloACP^Q31G/T35L^. ^1^H–^15^N correlation spectra were recorded for both proteins. For titration with Sfp R4–4, stocks of unlabeled Sfp (830 μM) were mixed with ^15^N-labeled ACP, 10% D_2_O and the appropriate amount of buffer, maintaining an ACP concentration of ∼100 μM throughout the titration with dilution effects never exceeded 10% and were accounted for during data analysis.

The averaged ^1^H and ^15^N weighted chemical shift perturbation (CSP) observed in ^1^H, ^15^N-HSQC spectra were calculated according to equation:^61^
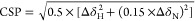


Here, Δδ_H_ is the ^1^H chemical shift difference, Δδ_N_ is the ^15^N chemical shift difference, and CSP is the averaged ^1^H and ^15^N weighted chemical shift difference in ppm.

The NMR backbone assignments of gloACP and gloACP^Q31G/T35L^ have been deposited in the in the BioMagResBank (www.bmrb.io) under the accession number 52726 and 52727 respectively.

### CS-Rosetta Modeling

Three-dimensional gloACP structural models were calculated with CS-Rosetta^39^ (version 2.01rev2019.06 using 40 CPUs within the NMRbox virtual environment)^62^ using experimental backbone ^13^CO, ^13^C^α^, ^13^C^β^, ^1^H^α^, ^1^H^N^ and ^15^N^H^ chemical shift restraints. Before setting up the CS-Rosetta run, the referenced gloACP backbone chemical shifts were submitted to TALOS-N^63^ for generation of dihedral restraints and predicting highly flexible or disordered regions. Since TALOS-N predicted highly dynamic amino acid stretches at the *N*- and *C*-termini of the gloACP construct the *N*-terminal purification tag (residue −19–0) and the last five C-terminal residues (76–80) were removed for the modeling procedure. To facilitate proper folding, repulsive energy was used for those residues during structure calculation. GloACP residues 54–58 were included in the calculation run although classified as dynamic by TALOS-N.

The CS-Rosetta run was considered to have converged based on the backbone C^α^-atom RMSD (root-mean-square deviation) < 2 Å between the lowest rescored energy structures. 1292 out of the 10,000 calculated structural models have a RMSD < 2 Å to the lowest energy model (Figure S12). The 10 lowest rescored energy models with a mean C^α^-RMSD of 0.84 ± 0.33 Å were chosen.

### *In Vitro* ACP Phosphopantetheinylation Assay

Standard phosphopantetheinylation assays of *apo*-ACPs (gloACP, panACP variants and dacACP variants) by Sfp were carried as previously described.^38,57^ In brief, 500 μL reactions were set up in 50 mM sodium phosphate buffer, pH 7.6 in glass vials at RT overnight with final concentrations of 150 μM ACP, 1 μM Sfp R4–4, 2.5 mM DTT, 1.5 mM CoA (lithium salt from CoALA Biosciences), and 10 mM MgCl_2_. Phosphopantetheinylation assays with *apo*-gloACP and gloPPT were set up under the same conditions, but with a gloPPT concentration of ∼2.5–10 μM and WT gloACP concentration of ∼200 μM. GloPPT phosphopantetheinylation reactions involving the addition of gloSCL and cofactors contained additional final concentrations of 3 μM gloSCL, 5 mM ATP (disodium trihydrate, Gold Biotechnology), and 0.5 mM sodium salicylate. The degree of conversion from *apo*-ACP into *holo*-ACP was assessed by LC-MS as described below.

### Analysis of ACPs by LC-MS

The loaded state of ACPs (*apo*, *holo*, *malonyl*, *salicyl*) of was analyzed by LC-MS (Table S3, Figures S4–S7, S9, S10, S15–S30, and S32).^64,65^ For LC-MS analysis, protein samples were diluted to ∼10 μM using HPLC grade water and analyzed using an Agilent Technologies InfinityLab G6125B LC/MS coupled with an Agilent 1260 Infinity II LC system loaded with a Waters XBridge Protein BEH C4 reverse phase column (300 Å, 3.55 μm, 2.1 mm × 50 mm) heated to 45 °C. Samples were analyzed via electrospray ionization mass spectrometry (ESI-MS) in the positive mode. The following gradient was used: 0–1 min 5% B; 1–3.1 min 95% B; 3.1–4.52 min 95% B; 4.52–4.92 min 5% B; 4.92–9 min 5% B (solvent A = water +0.1% (v/v) formic acid; solvent B = acetonitrile +0.1% (v/v) formic acid). ACPs (regardless of loaded state) eluted with a retention time of 4.2–4.6 min, as observed by absorbance at 254 and 280 nm as well as total ion count. Following data collection, mass spectra were deconvoluted using ESIprot online^66^ and plotted using Origin (Version 8.60. OriginLab Corporation, Northampton, MA). The observed deconvoluted MS was compared to the calculated *M*_*W*_ for ACPs in their various loaded states. The relative intensity of the *m*/*z* peaks assigned to *apo* versus *holo* state was used to approximate the percent conversion.

### Tandem Proteolysis Mass Spectrometry

Tandem proteolysis was performed on the SDS PAGE bands corresponding to the putative KS and CLF to confirm the protein identities by the Proteomics and Metabolomics Facility of the Wistar Institute located in Philadelphia, PA. Samples were reduced with tris (2-carboxyethyl) phosphine (TCEP), alkylated with iodoacetamide, and digested with trypsin. Resulting tryptic peptides were injected into a Waters ACQUITY UPLC Symmetry C18 trap column (100 Å, 180 μm × 2 mm packed with 5 μm C18 particles) and separated using their standard 90 min gradient formed by solvent A (water +0.1% (v/v) formic acid) and solvent B (acetonitrile +0.1% (v/v) formic acid). Peptides were scanned from 400 to 2000 *m*/*z* under positive ionization. The full scan was conducted at 70,000 resolution and data-dependent MS/MS scans at 175,000 resolution were performed on the resulting ions that exceeded a minimum threshold of 20,000. Peptide match was set as “preferred, exclude isotopes” and charge-state screening was enabled to reject singly and unassigned charged ions. The resulting peptide sequences were identified using MaxQuant 1.6.1.0 software (Figure S3).

### Malonylation of *holo*-gloACP^Q31G/T35L^

Malonylation protocols were adapted from Beltran-Alvarez et al.^67^ All reaction components were stored in 50 mM sodium phosphate buffer, pH 7.6. Prior to the addition of malonyl-CoA, *holo*-gloACP^Q31G/T35L^ and TCEP-HCl were mixed in a clear 2 mL glass vial and preincubated at 30 °C for 30 min without shaking. Final reaction concentrations were as follows: 75 μM *holo*-ACP, 1 mM TCEP-HCl, and 1 mM malonyl-CoA (lithium salt from CoALA Biosciences) in a total volume of 100 μL in 50 mM phosphate buffer, pH 7.6. For reactions involving EcFabD or ScFabD, these MCATs were added to reach final concentrations of 1.5 μM. Once all components were combined, reactions were incubated at 30 °C for 30 min prior to analysis of the ACP loaded state by LC-MS, as described above.

### Salicyl Priming of *holo*-gloACP^Q31G/T35L^

Salicyl priming of *holo*-gloACP^Q31G/T35L^ was carried out in 50 mM sodium phosphate buffer, pH 7.6. The protein was preincubated with 1 mM TCEP-HCl for 30 min at 30 °C in a clear 2 mL glass vial before adding additional components. Final reaction concentrations were as follows: 75 μM *holo*-gloACP^Q31G/T35L^ 1 mM TCEP-HCl, 3 μM gloSCL, 0.5 mM sodium salicylate, 5 mM MgCl_2_, and 5 mM ATP in a total volume of 200 μL. Once all components were combined, reactions were incubated at 30 °C for 30 min prior to analysis of the ACP loaded state by LC-MS, as described above.

### GloPKS In Vitro Reactions with Acetate Priming

*In vitro* reaction protocols were adapted from Cheng et al.^21^ All reaction components were stored in 50 mM sodium phosphate buffer (pH 7.6) with 10% (v/v) glycerol. Prior to the addition of gloKS-CLF, ScFabD and ^12/13^C malonyl-CoA, *holo*-gloACP^Q31G/T35L^ and TCEP-HCl were mixed in a clear 2 mL glass vial and preincubated at 30 °C for 30 min without shaking. Final reaction concentrations were as follows: 75 μM *holo*-ACP, 1 mM TCEP-HCl, 15 μM KS-CLF, 1.5 μM ScFabD, 10% (v/v) glycerol, and 2 mM ^12/13^C_3_-malonyl-CoA in a total volume of 300 μL. Once all components were combined, reactions were incubated at 30 °C for 16–18 h. The reaction was extracted (2 × 1 mL) with 99:1 EtOAc:AcOH. The extracts were combined, dried, resuspended in 300 μL MeOH, and analyzed by High-Res LC-MS (see below).

For large-scale reactions (100 × 1 mL or larger), malonyl-CoA was produced *in situ*, for a more cost-effective alternative to commercially available malonyl-CoA. Reactant stocks were prepared in the reaction buffer as follows: 150 μM *holo*-gloACP^Q31G/T35L^, 50 mM TCEP-HCl, 54 μM KS-CLF, 28 μM ScFabD, 309 μM malonyl-CoA synthetase (MatB, from *Rhizobium trifolii*), 250 mM MgCl_2_, 1 M sodium malonate (dibasic monohydrate from Sigma-Aldrich), 250 mM CoA, and 300 mM ATP. Reactions were set up in 100 × 1-dram clear glass vials in 1 mL volumes. First, *holo*-ACP (13.3 mL), TCEP-HCl (2 mL) and reaction buffer (34.6 mL) were combined in one conical tube and allowed to incubate for 30 °C for 30 min without shaking prior to aliquoting 500 μL into each of 100 × 1-dram clear glass vials. Next, in a second conical tube KS-CLF (18.5 mL), ScFabD (3.6 mL), MatB (6.5 mL), MgCl_2_ (2.8 mL) were combined prior to adding 314 μL into each reaction vial. This was followed by the addition of sodium malonate (100 μL), CoA (20 μL), and finally ATP (67 μL) into each of 100 reaction vials. Each 1 mL reaction was immediately mixed via pipetting. Final concentrations were as follows: 20 μM *holo*-ACP, 1 mM TCEP-HCl, 10 μM KS-CLF, 1 μM ScFabD, 20 μM MatB, 7 mM MgCl_2_, 100 mM sodium malonate, 5 mM CoA, and 20 mM ATP. All 100 × 1 mL reactions were incubated for 18 h at 30 °C, 80 rpm. Each 1 mL reaction was then extracted 3 × 5 mL of EtOAc:AcOH (99:1). Organic extracts were combined and dried under vacuum, and residual AcOH was removed using toluene azeotrope. Extracts were analyzed by High-Res LC-MS (see below).

### GloPKS In Vitro Reactions with Salicyl Priming

*In vitro* reaction protocols were adapted from Cheng et al.^21^ and run in 50 mM sodium phosphate buffer, pH 7.6. In brief, *holo*-gloACP^Q31G/T35L^ and TCEP-HCl were incubated for 30 min at 30 °C followed by the sequential addition of gloSCL, sodium salicylate, MgCl_2_, and ATP. After an additional 30 min incubation at 30 °C, ScFabD, gloKS-CLF, glycerol, and ^12/13^C_3_-malonyl-CoA were added and all components were incubated for 3 h at 30 °C. Final reaction concentrations were as follows: 75 μM *holo*-gloACP, 1 mM TCEP-HCl, 1.5 μM gloSCL, 0.5 mM sodium salicylate, 5 mM MgCl_2_, 5 mM ATP, 15 μM gloKS-CLF, 1.5 μM FabD, 10% (v/v) glycerol, and 2 mM ^12/13^C_3_-malonyl-CoA in a total volume of 300 μL. The reaction was extracted (2 × 1 mL) of 99:1 EtOAc:AcOH. The extracts were combined, dried, resuspended in 300 μL MeOH, and analyzed by High-Res LC-MS (see below).

### Characterization of In Vitro Reaction Products by High-Resolution LC-MS

Molecules were analyzed on a Waters Acquity I-Class UPLC system coupled to a Synapt G2Si HDMS mass spectrometer in positive ion mode with a heated electrospray ionization (ESI) source in a Z-spray configuration. For small molecules, LC separation was performed on a Waters Acquity UPLC BEH C18 (130 Å, 2.1 mm × 50 mm packed with 1.7 μm C18 particles) using a 0.6 mL/min gradient of 95/5 to 15/85 A/B over the course of 4 min. Eluent A is 0.1% (v/v) formic acid in water and B is 0.1% (v/v) formic acid in acetonitrile. Conditions on the mass spectrometer were as follows: capillary voltage 0.5 kV, sampling cone 40 V, source offset 80 V, source 120 °C, desolvation 250 °C, cone gas 0 L/h, desolvation gas 1000 L/h and nebulizer 6.5 bar. The analyzer was operated in resolution mode and low energy data were collected between 100 and 2000 Da at 0.2 s scan time. Data were collected using a 20–40 V ramp trap collision energy. Masses were extracted from the TOF MS TICs using a 0.01 or 0.05 Da abs width.

## Supporting Information Available

Additional experimental methods; comparative gene cluster analysis; gene and protein sequences; protein characterization including SDS PAGE, Western Blot, tandem proteolysis mass spectrometry; LCMS spectra of ACPs; protein NMR and models; multiple sequence alignments; Figures S1–S33; Tables S1–S3 (PDF)

## References

[ref1] Scott T. A.; Piel J. The Hidden Enzymology of Bacterial Natural Product Biosynthesis. Nat. Rev. Chem. 2019, 3 (7), 404–425. 10.1038/s41570-019-0107-1. 32232178 PMC7104373

[ref2] Walsh C. T.; Fischbach M. A. Natural Products Version 2.0: Connecting Genes to Molecules. J. Am. Chem. Soc. 2010, 132 (8), 2469–2493. 10.1021/ja909118a. 20121095 PMC2828520

[ref3] Cortes J.; Haydock S. F.; Roberts G. A.; Bevitt D. J.; Leadlay P. F. An Unusually Large Multifunctional Polypeptide in the Erythromycin-Producing Polyketide Synthase of Saccharopolyspora Erythraea. Nature 1990, 348 (6297), 176–178. 10.1038/348176a0. 2234082

[ref4] Donadio S.; Staver M. J.; McAlpine J. B.; Swanson S. J.; Katz L. Modular Organization of Genes Required for Complex Polyketide Biosynthesis. Science 1991, 252 (5006), 675–679. 10.1126/science.2024119. 2024119

[ref5] Khosla C.; Herschlag D.; Cane D. E.; Walsh C. T. Assembly Line Polyketide Synthases: Mechanistic Insights and Unsolved Problems. Biochemistry 2014, 53 (18), 2875–2883. 10.1021/bi500290t. 24779441 PMC4020578

[ref6] Rivers M. A. J.; Lowell A. N. Expanding the Biosynthetic Toolbox: The Potential and Challenges of In Vitro Type II Polyketide Synthase Research. SynBio 2024, 2 (1), 85–111. 10.3390/synbio2010006.

[ref7] Hertweck C.; Luzhetskyy A.; Rebets Y.; Bechthold A. Type II Polyketide Synthases: Gaining a Deeper Insight into Enzymatic Teamwork. Nat. Prod. Rep. 2007, 24 (1), 162–190. 10.1039/B507395M. 17268612

[ref8] Stevens D. C.; Conway K. R.; Pearce N.; Villegas-Peñaranda L. R.; Garza A. G.; Boddy C. N. Alternative Sigma Factor Over-Expression Enables Heterologous Expression of a Type II Polyketide Biosynthetic Pathway in Escherichia Coli. PLoS One 2013, 8 (5), e64858–e64858 10.1371/journal.pone.0064858. 23724102 PMC3665592

[ref9] Zhang W.; Li Y.; Tang Y. Engineered Biosynthesis of Bacterial Aromatic Polyketides in Escherichia Coli. Proc. Natl. Acad. Sci. U. S. A. 2008, 105 (52), 20683–20688. 10.1073/pnas.0809084105. 19075227 PMC2634872

[ref10] Keatinge-Clay A. T.; Maltby D. A.; Medzihradszky K. F.; Khosla C.; Stroud R. M. An Antibiotic Factory Caught in Action. Nat. Struct. Mol. Bio.l 2004, 11 (9), 888–893. 10.1038/nsmb808. 15286722

[ref11] Carreras C. W.; Khosla C. Purification and in Vitro Reconstitution of the Essential Protein Components of an Aromatic Polyketide Synthase. Biochemistry 1998, 37 (8), 2084–2088. 10.1021/bi972919. 9518007

[ref12] Carreras C. W.; Pieper R.; Khosla C. Efficient Synthesis of Aromatic Polyketides In Vitro by the Actinorhodin Polyketide Synthase. J. Am. Chem. Soc. 1996, 118 (21), 5158–5159. 10.1021/ja960479o.

[ref13] Matharu A. -L.; Cox R. J.; Crosby J.; Byrom K. J.; Simpson T. J. MCAT Is Not Required for in Vitro Polyketide Synthesis in a Minimal Actinorhodin Polyketide Synthase from Streptomyces Coelicolor. Chem. Biol. 1998, 5 (12), 699–711. 10.1016/s1074-5521(98)90663-9. 9862793

[ref14] McDaniel R.; Khosla S. E.; Hopwood D. A.; Khosla C. Engineered Biosynthesis of Novel Polyketides: Manipulation and Analysis of an Aromatic Polyketide Synthase with Unproven Catalytic Specificities. J. Am. Chem. Soc. 1993, 115 (25), 11671–11675. 10.1021/ja00078a002.

[ref15] McDaniel R.; Ebert-Khosla S.; Hopwood D. A.; Khosla C. Rational Design of Aromatic Polyketide Natural Products by Recombinant Assembly of Enzymatic Subunits. Nature 1995, 375 (6532), 549–554. 10.1038/375549a0. 7791871

[ref16] Fu H.; Khosla S. E.; Hopwood D. A.; Khosla C. Engineered Biosynthesis of Novel Polyketides: Dissection of the Catalytic Specificity of the act Ketoreductase. J. Am. Chem. Soc. 1994, 116 (10), 4166–4170. 10.1021/ja00089a003.

[ref17] Fu H.; Ebert-Khosla S.; Hopwood D. A.; Khosla C. Relaxed Specificity of the Oxytetracycline Polyketide Synthase for an Acetate Primer in the Absence of a Malonamyl Primer. J. Am. Chem. Soc. 1994, 116 (14), 6443–6444. 10.1021/ja00093a058.

[ref18] Shen B.; Summers R. G.; Wendt-Pienkowski E.; Hutchinson C. R. The Streptomyces Glaucescens tcmKL Polyketide Synthase and tcmN Polyketide Cyclase Genes Govern the Size and Shape of Aromatic Polyketides. J. Am. Chem. Soc. 1995, 117 (26), 6811–6821. 10.1021/ja00131a002.

[ref19] Shen B.; Hutchinson C. R. Deciphering the Mechanism for the Assembly of Aromatic Polyketides by a Bacterial Polyketide Synthase. Proc. Natl. Acad. Soc. U. S. A. 1996, 93 (13), 6600–6604. 10.1073/pnas.93.13.6600. PMC39071 8692863

[ref20] Shen B.; Hutchinson C. R. Enzymatic Synthesis of a Bacterial Polyketide from Acetyl and Malonyl Coenzyme A. Science 1993, 262 (5139), 1535–1540. 10.1126/science.8248801. 8248801

[ref21] Cheng Q.; Xiang L.; Izumikawa M.; Meluzzi D.; Moore B. S. Enzymatic Total Synthesis of Enterocin Polyketides. Nat. Chem. Biol. 2007, 3 (9), 557–558. 10.1038/nchembio.2007.22. 17704772

[ref22] Shen Y.; Yoon P.; Yu T. W.; Floss H. G.; Hopwood D.; Moore B. S. Ectopic Expression of the Minimal whiE Polyketide Synthase Generates a Library of Aromatic Polyketides of Diverse Sizes and Shapes. Proc. Natl. Acad. Sci. U. S. A. 1999, 96 (7), 3622–3627. 10.1073/pnas.96.7.3622. 10097087 PMC22344

[ref23] Xu Z.; Schenk A.; Hertweck C. Molecular Analysis of the Benastatin Biosynthetic Pathway and Genetic Engineering of Altered Fatty Acid–Polyketide Hybrids. J. Am. Chem. Soc. 2007, 129 (18), 6022–6030. 10.1021/ja069045b. 17439117

[ref24] Tang Y.; Lee T. S.; Khosla C.; G. Matthews R. Engineered Biosynthesis of Regioselectively Modified Aromatic Polyketides Using Bimodular Polyketide Synthases. PloS Biol. 2004, 2 (2), 0227–0238. 10.1371/journal.pbio.0020031. PMC340942 14966533

[ref25] Ridley C. P.; Lee H. Y.; Khosla C. Evolution of Polyketide Synthases in Bacteria. Proc. Natl. Acad. Soc. U. S. A. 2008, 105 (12), 4595–4600. 10.1073/pnas.0710107105. PMC2290765 18250311

[ref26] Hillenmeyer M. E.; Vandova G. A.; Berlew E. E.; Charkoudian L. K. Evolution of Chemical Diversity by Coordinated Gene Swaps in Type II Polyketide Gene Clusters. Proc. Natl. Acad. Soc. U. S. A. 2015, 112 (45), 13952–13957. 10.1073/pnas.1511688112. PMC4653136 26499248

[ref27] McBride C. M.; Miller E. L.; Charkoudian L. K. An Updated Catalogue of Diverse Type II Polyketide Synthase Biosynthetic Gene Clusters Captured from Large-Scale Nucleotide Databases. Microb. Genomics 2023, 9 (3), mgen000965 10.1099/mgen.0.000965. PMC10132072 36951894

[ref28] Cummings M.; Peters A. D.; Whitehead G. F. S.; Menon B. R. K.; Micklefield J.; Webb S. J.; Takano E.; Id G. F. S. W.; Khosla C. Assembling a Plug-and-Play Production Line for Combinatorial Biosynthesis of Aromatic Polyketides in Escherichia Coli. PloS Biol. 2019, 17 (7), e3000347 10.1371/journal.pbio.3000347. 31318855 PMC6638757

[ref29] Klein J. G.; Wu Y.; Kokona B.; Charkoudian L. K. Widening the Bottleneck: Heterologous Expression, Purification, and Characterization of the Ktedonobacter Racemifer Minimal Type II Polyketide Synthase in Escherichia Coli. Bioorg. Med. Chem. 2020, 28 (20), 115686–115686. 10.1016/j.bmc.2020.115686. 33069071

[ref30] Liu X.; Hua K.; Liu D.; Wu Z.-L.; Wang Y.; Zhang H.; Deng Z.; Pfeifer B. A.; Jiang M. Heterologous Biosynthesis of Type II Polyketide Products Using E. Coli. ACS Chem. Biol. 2020, 15 (5), 1177–1183. 10.1021/acschembio.9b00827. 31825590

[ref31] Bräuer A.; Zhou Q.; Grammbitter G. L. C.; Schmalhofer M.; Rühl M.; Kaila V. R. I.; Bode H. B.; Groll M. Structural Snapshots of the Minimal PKS System Responsible for Octaketide Biosynthesis. Nat. Chem. 2020, 12, 755–763. 10.1038/s41557-020-0491-7. 32632186

[ref32] Crosby J.; Crump M. P. The Structural Role of the Carrier Protein – Active Controller or Passive Carrier. Nat. Prod. Rep. 2012, 29 (10), 1111–1137. 10.1039/c2np20062g. 22930263

[ref33] Sunbul M.; Marshall N. J.; Zou Y.; Zhang K.; Yin J. Catalytic Turnover-Based Phage Selection for Engineering the Substrate Specificity of Sfp Phosphopantetheinyl Transferase. J. Mol. Biol. 2009, 387 (4), 883–898. 10.1016/j.jmb.2009.02.010. 19340948

[ref34] Beld J.; Sonnenschein E. C.; Vickery C. R.; Noel J. P.; Burkart M. D. The Phosphopantetheinyl Transferases: Catalysis of a Post-Translational Modification Crucial for Life. Nat. Prod. Rep. 2014, 31 (1), 61–108. 10.1039/C3NP70054B. 24292120 PMC3918677

[ref35] Nordberg H.; Cantor M.; Dusheyko S.; Hua S.; Poliakov A.; Shabalov I.; Smirnova T.; Grigoriev I. V.; Dubchak I. The Genome Portal of the Department of Energy Joint Genome Institute: 2014 Updates. Nucleic Acids Res. 2014, 42, D26–D31. 10.1093/nar/gkt1069. 24225321 PMC3965075

[ref36] Pfeifer B. A.; Admiraal S. J.; Gramajo H.; Cane D. E.; Khosla C. Biosynthesis of Complex Polyketides in a Metabolically Engineered Strain of E. Coli. Science 2001, 291 (5509), 1790–1792. 10.1126/science.1058092. 11230695

[ref37] Quadri L. E.; Weinreb P. H.; Lei M.; Nakano M. M.; Zuber P.; Walsh C. T. Characterization of Sfp, a Bacillus Subtilis Phosphopantetheinyl Transferase for Peptidyl Carrier Protein Domains in Peptide Synthetases. Biochemistry 1998, 37 (6), 1585–1595. 10.1021/bi9719861. 9484229

[ref38] Lambalot R. H.; Gehring A. M.; Flugel R. S.; Zuber P.; LaCelle M.; Marahiel M. A.; Reid R.; Khosla C.; Walsh C. T. A New Enzyme Superfamily — the Phosphopantetheinyl Transferases. Chem. Biol. 1996, 3 (11), 923–936. 10.1016/S1074-5521(96)90181-7. 8939709

[ref39] Shen Y.; Lange O.; Delaglio F.; Rossi P.; Aramini J. M.; Liu G.; Eletsky A.; Wu Y.; Singarapu K. K.; Lemak A.; Ignatchenko A.; Arrowsmith C. H.; Szyperski T.; Montelione G. T.; Baker D.; Bax A. Consistent Blind Protein Structure Generation from NMR Chemical Shift Data. Proc. Natl. Acad. Sci. U. S. A. 2008, 105 (12), 4685–4690. 10.1073/pnas.0800256105. 18326625 PMC2290745

[ref40] Milligan J. C.; Lee D. J.; Jackson D. R.; Schaub A. J.; Beld J.; Barajas J. F.; Hale J. J.; Luo R.; Burkart M. D.; Tsai S.-C. Molecular Basis for Interactions between an Acyl Carrier Protein and a Ketosynthase. Nat. Chem. Biol. 2019, 15 (7), 669–671. 10.1038/s41589-019-0301-y. 31209348 PMC7323458

[ref41] Nguyen C.; Haushalter R. W.; Lee D. J.; Markwick P. R. L.; Bruegger J.; Caldara-Festin G.; Finzel K.; Jackson D. R.; Ishikawa F.; O’Dowd B.; McCammon J. A.; Opella S. J.; Tsai S. C.; Burkart M. D. Trapping the Dynamic Acyl Carrier Protein in Fatty Acid Biosynthesis. Nature 2014, 505, 427–431. 10.1038/nature12810. 24362570 PMC4437705

[ref42] Dell M.; Tran M. A.; Capper M. J.; Sundaram S.; Fiedler J.; Koehnke J.; Hellmich U. A.; Hertweck C. Trapping of a Polyketide Synthase Module after C–C Bond Formation Reveals Transient Acyl Carrier Domain Interactions. Angew. Chem., Int. Ed. 2024, 63 (9), e202315850 10.1002/anie.202315850. 38134222

[ref43] Tufar P.; Rahighi S.; Kraas F. I.; Kirchner D. K.; Lohr F. N.; Henrich E.; Köpke J.; Dikic I.; Güntert P.; Marahiel M. A.; Dötsch V. Crystal Structure of a PCP/Sfp Complex Reveals the Structural Basis for Carrier Protein Posttranslational Modification. Chem. Biol. 2014, 21 (4), 552–562. 10.1016/j.chembiol.2014.02.014. 24704508

[ref44] Yang D.; Jang W. D.; Lee S. Y. Production of Carminic Acid by Metabolically Engineered Escherichia Coli. J. Am. Chem. Soc. 2021, 143 (14), 5364–5377. 10.1021/jacs.0c12406. 33797895

[ref45] Florova G.; Kazanina G.; Reynolds K. A. Enzymes Involved in Fatty Acid and Polyketide Biosynthesis in Streptomyces Glaucescens: Role of FabH and FabD and Their Acyl Carrier Protein Specificity. Biochemistry 2002, 41 (33), 10462–10471. 10.1021/bi0258804. 12173933

[ref46] Serre L.; Verbree E. C.; Dauter Z.; Stuitje A. R.; Derewenda Z. S. The Escherichia Coli Malonyl-CoA: Acyl Carrier Protein Transacylase at 1.5-Å Resolution.: Crystal structure of a fatty acid synthase component. J. Biol. Chem. 1995, 270 (22), 12961–12964. 10.1074/jbc.270.22.12961. 7768883

[ref47] Arthur C. J.; Szafranska A.; Evans S. E.; Findlow S. C.; Burston S. G.; Owen P.; Clark-Lewis I.; Simpson T. J.; Crosby J.; Crump M. P. Self-Malonylation Is an Intrinsic Property of a Chemically Synthesized Type II Polyketide Synthase Acyl Carrier Protein. Biochemistry 2005, 44 (46), 15414–15421. 10.1021/bi051499i. 16285746

[ref48] Aragozzini F.; Craveri R.; Maconi E.; Ricca G. S.; Scolastico C. Thermorubin Biosynthesis: Evidence for the Involvement of Both Salicylic Acid and an Undecaketide. J. Chem. Soc., Perkin Trans. 1 1988, 1988 (7), 1865–1867. 10.1039/p19880001865.

[ref49] McCord J. P.; Kohanov Z. A.; Lowell A. N. Thermorubin Biosynthesis Initiated by a Salicylate Synthase Suggests an Unusual Conversion of Phenols to Pyrones. ACS Chem. Biol. 2022, 17 (11), 3169–3177. 10.1021/acschembio.2c00606. 36255735

[ref50] Buyachuihan L.; Stegemann F.; Grininger M. How Acyl Carrier Proteins (ACPs) Direct Fatty Acid and Polyketide Biosynthesis. Angew. Chem., Int. Ed. 2024, 63 (4), e202312476 10.1002/anie.202312476. 37856285

[ref51] Leblanc C.; Prudhomme T.; Tabouret G.; Ray A.; Burbaud S.; Cabantous S.; Mourey L.; Guilhot C.; Chalut C. 4′-Phosphopantetheinyl Transferase PptT, a New Drug Target Required for Mycobacterium Tuberculosis Growth and Persistence In Vivo. PloS Pathog. 2012, 8 (12), e1003097 10.1371/journal.ppat.1003097. 23308068 PMC3534377

[ref52] Saha S. K.; Uma L.; Subramanian G. An Improved Method for Marine Cyanobacterial DNA Isolation. World J. Microbiol. Biotechnol. 2005, 21 (6), 877–881. 10.1007/s11274-004-6187-0.

[ref53] Blin K.; Shaw S.; Steinke K.; Villebro R.; Ziemert N.; Lee S. Y.; Medema M. H.; Weber T. antiSMASH 5.0: Updates to the secondary metabolite genome mining pipeline. Nucleic Acids Res. 2019, 47 (W1), W81–W87. 10.1093/nar/gkz310. 31032519 PMC6602434

[ref54] Gibson D. G.; Young L.; Chuang R. Y.; Venter J. C.; Hutchison C. A. 3.; Smith H. O. Enzymatic Assembly of DNA Molecules up to Several Hundred Kilobases. Nat. Methods 2009, 6 (5), 343–345. 10.1038/nmeth.1318. 19363495

[ref55] Robert X.; Gouet P. Deciphering Key Features in Protein Structures with the New ENDscript Server. Nucleic Acids Res. 2014, 42 (W1), W320–W324. 10.1093/nar/gku316. 24753421 PMC4086106

[ref56] Sievers F.; Higgins D. G. Clustal Omega. Curr. Proctoc. Bioinformatics 2014, 2014, 3–13. 10.1002/0471250953.bi0313s48. 25501942

[ref57] Zhang W.; Tang Y. In Vitro Analysis of Type II Polyketide Synthase. In Methods in Enzymology; Elsevier, 2009; Vol. 459, pp. 367–393. DOI: 10.1016/S0076-6879(09)04616-3. 19362648

[ref58] Azatian S. B.; Kaur N.; Latham M. P. Increasing the Buffering Capacity of Minimal Media Leads to Higher Protein Yield. J. Biol. NMR 2019, 73 (1), 11–17. 10.1007/s10858-018-00222-4. PMC6441617 30613903

[ref59] Wishart D. S.; Bigam C. G.; Yao J.; Abildgaard F.; Dyson H. J.; Oldfield E.; Markley J. L.; Sykes B. D. 1H,13C and 15N Chemical Shift Referencing in Biomolecular NMR. J. Biol. NMR 1995, 6 (2), 135–140. 10.1007/BF00211777. 8589602

[ref60] Skinner S. P.; Fogh R. H.; Boucher W.; Ragan T. J.; Mureddu L. G.; Vuister G. W. CcpNmr AnalysisAssign: A Flexible Platform for Integrated NMR Analysis. J. Biol. NMR 2016, 66 (2), 111–124. 10.1007/s10858-016-0060-y. PMC5095159 27663422

[ref61] Williamson M. P. Using Chemical Shift Perturbation to Characterise Ligand Binding. Prog. Nucl. Magn. Reson. Spectrosc. 2013, 73, 1–16. 10.1016/j.pnmrs.2013.02.001. 23962882

[ref62] Maciejewski M. W.; Schuyler A. D.; Gryk M. R.; Moraru I. I.; Romero P. R.; Ulrich E. L.; Eghbalnia H. R.; Livny M.; Delaglio F.; Hoch J. C. NMRbox: A Resource for Biomolecular NMR Computation. Biophys. J. 2017, 112 (8), 1529–1534. 10.1016/j.bpj.2017.03.011. 28445744 PMC5406371

[ref63] Shen Y.; Bax A. Protein Backbone and Sidechain Torsion Angles Predicted from NMR Chemical Shifts Using Artificial Neural Networks. J. Biol. NMR 2013, 56 (3), 227–241. 10.1007/s10858-013-9741-y. PMC3701756 23728592

[ref64] Wang Y.-Y.; Zhang X.-S.; Luo H.-D.; Ren N.-N.; Jiang X.-H.; Jiang H.; Li Y.-Q. Characterization of Discrete Phosphopantetheinyl Transferases in Streptomyces Tsukubaensis L19 Unveils a Complicate Phosphopantetheinylation Network. Sci. Rep. 2016, 6 (1), 24255 10.1038/srep24255. 27052100 PMC4823652

[ref65] Beld J.; Finzel K.; Burkart M. D. Versatility of Acyl-Acyl Carrier Protein Synthetases. Chem. Biol. 2014, 21 (10), 1293–1299. 10.1016/j.chembiol.2014.08.015. 25308274 PMC4224610

[ref66] Winkler R. ESIprot: A Universal Tool for Charge State Determination and Molecular Weight Calculation of Proteins from Electrospray Ionization Mass Spectrometry Data. Rapid Commun. Mass Spectrom. 2010, 24 (3), 285–294. 10.1002/rcm.4384. 20049890

[ref67] Beltran-Alvarez P.; Cox R. J.; Crosby J.; Simpson T. J. Dissecting the Component Reactions Catalyzed by the Actinorhodin Minimal Polyketide Synthase. Biochemistry 2007, 46 (50), 14672–14681. 10.1021/bi701784c. 18034463

